# Beneficial Effects of *Clitoria ternatea* Flower and *Citrus limon* Fruit Beverage on Nutritional Status, Lipid Profile, and Adipokine Parameters on Male Rats With Obesity

**DOI:** 10.1155/jobe/6664514

**Published:** 2026-01-07

**Authors:** Fista Utami, Dono Indarto, Shanti Listyawati

**Affiliations:** ^1^ Master Program of Nutrition Sciences, Universitas Sebelas Maret, Jl. Ir Sutami No. 36, Surakarta, Jawa Tengah, 57126, Indonesia, uns.ac.id; ^2^ Department of Nutrition, Faculty of Sports and Health Sciences, Universitas Negeri Surabaya, Jl. Lidah Wetan, Surabaya, Jawa Timur, 60231, Indonesia, unesa.ac.id; ^3^ Department of Physiology and Biomedical Laboratory, Faculty of Medicine, Universitas Sebelas Maret, Jl. Ir Sutami No. 36, Surakarta, Jawa Tengah, 57126, Indonesia, uns.ac.id; ^4^ Doctoral Program of Medical Sciences, Faculty of Medicine, Universitas Sebelas Maret, Jl. Ir Sutami No. 36, Surakarta, Jawa Tengah, 57126, Indonesia, uns.ac.id; ^5^ Biology Department, Faculty of Mathematics and Natural Sciences, Universitas Sebelas Maret, Jl. Ir Sutami No. 36, Surakarta, Jawa Tengah, 57126, Indonesia, uns.ac.id

**Keywords:** anti-obesity, body weight, DPP4 activity, herbal beverage, leptin levels, lipid profile

## Abstract

**Background:**

Butterfly pea flowers (*Clitoria ternatea* L.) and lemon fruits (*Citrus limon*) are rich in phytochemicals and have shown potential anti‐obesity effects. However, the combination of these two medicinal plants as an herbal beverage has not been extensively studied.

**Objective:**

This study aimed to evaluate the effects of an herbal beverage composed of butterfly pea flowers and lemon fruits (BPL) on body weight (BW), Lee index (LI), body fat content (BFC), lipid profile (total cholesterol, low‐density lipoprotein‐cholesterol [LDL‐C], high‐density lipoprotein‐cholesterol [HDL‐C], and triglycerides [TGs]), leptin levels, and DPP4 activity in obese male Wistar rats.

**Methods:**

A pre‐ and post‐test experimental design was conducted using 40 obese male Wistar rats, divided into five groups: NC (negative control, given 3 mL/day mineral water), positive control (PC, given 3 mL/day plant stanol ester), BPL1 received 3 mL/day BPL 75:25%, BPL2 received 3 mL/day BPL 80:20%, and BPL3 received 3 mL/day BPL 85:15%. All treatments were administered orally via gastric probe for 21 days. Data were analyzed using appropriate statistical tests to assess significant differences.

**Results:**

The BPL3 group showed the greatest reduction in BW and LI. The BPL1 group had the greatest reduction in TG levels, followed by the BPL3 group. The greatest reduction in leptin levels was found in the BPL2 group, followed by the BPL3 group.

**Conclusion:**

Oral administration of BPL herbal beverage with 85:15% formulation reduces BW and LI through the modulation of lipid metabolism and hormonal regulation.

## 1. Introduction

Obesity is an excessive accumulation of body fat with a body mass index (BMI) level of ≥ 30 kg/m^2^ that can increase the risk of degenerative diseases. Over 43% of adults were obese in 2022 worldwide and are estimated to reach 50% in 2030 [1]. High consumption of ultraprocessed foods (UPFs), which are high in sugar, salt, and fats, and low in nutritional value, is strongly associated with obesity and metabolic disorders [2]. The imbalance between energy intake and expenditure leads to obesity and metabolic changes, such as dyslipidemia, resulting in a reduction in adiponectin levels, an increase in leptin levels, and fatty acids release into the blood circulation [3]. Increased nonesterified fatty acids (NEFAs) in blood circulation also cause insulin resistance, hyperglycemia, and dyslipidemia that lead to Type 2 diabetes mellitus, hypertension, heart failure, and cancer [4].

Dipeptidyl Peptidase 4 (DPP4) is a serine protease that degrades incretin hormones like glucagon‐like peptide‐1, which are crucial for insulin secretion and glucose homeostasis. Elevated DPP4 activity is frequently observed in obesity and contributes to impaired glucose tolerance, increased inflammation, and metabolic dysfunction [5, 6]. In vitro and in vivo studies showed that DPP4 expression in subcutaneous and visceral adipose tissues positively correlated with BMI, adipose cell number, size, and inflammation [7]. Furthermore, circulating levels of soluble DPP4 were higher in obese patients compared to lean individuals of the same age. The adipose tissue biopsies revealed that DPP4 expression is influenced by total fat mass and adipose tissue distribution [6, 8]. Therefore, the inhibition of DPP4 expression and activity is another way to reduce body weight (BW) in obese individuals.

Diet management for obese patients involves a low‐energy diet and increased physical activity, which has been successful in BW reduction. This diet has almost the same principles as balanced nutrition, but the calories are decreased by about 500–750 kcal/day (or ∼30% reduction) from the daily requirement for the weight loss of around 0.5 kg/week [9]. However, a qualitative study found that individuals with Class III obesity frequently failed to follow prescribed very low‐energy diets, primarily due to social eating, emotional triggers, and unplanned deviations from the diet. Some study participants reported that they consumed more than 100% of their daily caloric requirements [10]. Diet‐induced obesity, such as high intake of fats, carbohydrates, fructose, and sucrose, contributes to leptin resistance, which weakens its anorexigenic effect and perpetuates overeating [11].

Natural sources that are high in flavonoids can be potentially used to treat obesity, such as butterfly pea flowers and lemon fruits. Butterfly pea flower is known as an edible flower that has high bioactive compounds such as anthocyanins, alkaloids, flavonoids, terpenoids, tannins, saponins, and phenols [12]. A previous study stated that administration of 1000 mg/kg BW dried butterfly pea flower extract decreased BW in male Swiss Albino rats with a stress model [13]. Delphinidin is a derivative of anthocyanin in the butterfly pea flower that can activate adenosine monophosphate protein kinase (AMPK) to increase the adipose triglyceride (TG) lipase activity. This enzyme plays a role in the catabolism of fatty acids into energy and the body’s lipolysis process [14]. A systematic review reported that butterfly pea flowers potentially prevent weight gain and induce weight loss by reducing the expression of adipogenicity genes and activating the lipase [15].

In addition to the butterfly pea flower benefits, lemon fruits contain high bioactive compounds such as hesperidin, citric acid, and phenols [16]. A study used a lemon fermented product with *Lactobacillu*s OPC1 supplementation, resulting in a 9.7% reduction in BW and a 25.7% decrease in fat tissue mass in Wistar rats fed a high‐fat diet (HFD) [17]. Hesperidin also increases the expression of AMPK for lipolysis, stimulates cholecystokinin release, and decreases appetite [18]. A previous study also showed that Berastagi orange peel extract significantly improved lipid metabolism by reducing low‐density lipoprotein‐cholesterol (LDL‐C), TG, and DPP4 activity while increasing high‐density lipoprotein‐cholesterol (HDL‐C) in male obese rats [19]. Since DPP4 is a key enzyme in incretin metabolism and inflammation, its inhibition represents a promising anti‐obesity strategy. Ten natural supplements showed wide variability in the citrus bioflavonoid content, including hesperidin, which potentially became DPP‐4 inhibitors (1.9–400 mg rutin equivalents/day) [20]. This bioactive variability indicates that differences in their composition and bioavailability strongly influence DPP‐4 inhibition.

So far, the combination of butterfly pea flower and lemon fruits has not been reported in experimental animal models of obesity. Lemon fruits have an acidic pH that is known to enhance the stability of anthocyanin pigments, particularly those derived from the butterfly pea flower [21]. One research shows that anthocyanins from butterfly pea exhibit the highest stability and antioxidant activity at low to moderate acidic conditions (pH 3.6–5.4), while stability deteriorates significantly in neutral to alkaline pH [22]. Our recent study showed that combining butterfly pea flowers and lemon fruits enhances total flavonoids, anthocyanins, and antioxidant levels. It can potentially be developed as a herbal beverage to support low‐energy diets in obesity management [15]. Meanwhile, another study in Indonesia found that consuming 2 g/day of plant stanol esters (PSEs) led to a 5.7% reduction in total cholesterol and a 9.3% decrease in LDL after two to 4 weeks [23]. PSE may slightly lower TGs and fasting blood glucose levels, which also indicates potential anti‐obesity benefits [24]. Therefore, the PSE beverage was used for the positive control (PC) group in our research study. Furthermore, leptin levels and DPP4 activity become important biomarkers for the weight‐lowering effect of BPL beverages [7, 8]. Therefore, this study aims to investigate the effects of the BPL herbal beverage on BW, LI, body fat content (BFC) percentage, lipid profiles, leptin levels, and DPP4 activity in male rats with obesity.

## 2. Materials and Methods

### 2.1. Materials

Dried butterfly pea flowers were purchased from a farmer in Kampung BW Pringsewu Regency, Lampung Province, Indonesia, while fresh lemon fruits were purchased from a farmer in Batu City, East Java Province, Indonesia. A standard rat feed (BR2) was purchased from PT Japfa Comfeed Indonesia Tbk, Sragen Regency, Central Java Province, Indonesia. Mineral water was obtained from Danone Group PT Aqua Golden Mississippi, Indonesia, with registration number MD 265210004169. The PSE beverage used in this study was obtained from an Indonesian commercial product for human consumption by PT Kalbe Nutritionals, a subsidiary of Kalbe Farma Group, Jakarta. 100 mL of the PSE beverage contained 50 kcal, 1.7 g PSE, 2 g fats, 1 g proteins, and 7 g total carbohydrates, with isomaltose and sucralose sweeteners. CV Dunia Kaca, Karanganyar Regency, Central Java, Indonesia, provided male Wistar rats with B‐2316/IPH.1/KS.02.03/VI/2019 animal strain identification number and 313/SKKH/III/2022 animal health identification number. Only male rats were used in this study to minimize potential variability due to hormonal fluctuations related to the estrous cycle in female rats. In addition, biological sex differences can influence metabolic responses, lipid profiles, and adipokine levels, such as leptin [25].

### 2.2. Preparation of BPL Herbal Beverages

BPL beverage was developed based on the formulation from a previous study, which had been registered at the Directorate General of Intellectual Property, Indonesian Ministry of Law, with S00202214250 registration number [26]. In general, the dried butterfly pea flowers were soaked in 60°C boiled water with a 1:100 (weight/volume) ratio for 15 min. Fresh lemon fruits were squeezed using a squeezer to get lemon juice. Furthermore, brewed butterfly pea flowers were mixed with lemon juice to make beverages with BPL1 (75:25% ratio), BPL2 (80:20% ratio), and BPL3 (85:15% ratio). The BPL herbal beverages were then stored in dark bottles before further analysis. Table 1 indicates that the BPL formulation ratios were selected based on preliminary testing to identify the optimal combination that yields the highest total flavonoid and anthocyanin contents [15]. Total flavonoid content was determined using the colorimetric method, while total anthocyanin levels were determined by the pH differential method. In this study, the beverage was prepared fresh daily to maintain the stability of bioactive compounds during treatment periods. After preparation, the beverage was stored in a dark bottle to minimize light exposure and administered within 2 h of preparation.

**Table 1:** Total flavonoids and anthocyanins levels in BPL1–BPL3.

Formulations	Total flavonoids (ppm)	Anthocyanins (ppm)
BPL1	993.50 ± 6.36	424.41 ± 25.75
BPL2	1.148.00 ± 7.07	443.42 ± 23.78
BPL3	1.265.00 ± 21.92	485.38 ± 22.08

### 2.3. Study Design of Wistar Rat Experiment

This experimental laboratory used male Wistar rats with a pre–post‐test control group design, conducted from March to August 2022. The protocol of this study was approved by the Research Ethics Committee, Faculty of Medicine, Universitas Sebelas Maret, Surakarta, Indonesia (Protocol ID 01/02/02/2022/10). The inclusion criteria for this study were male rats aged 8–9 weeks, weighing 150–200 g, and in good health with active movements. The sample size was calculated using a formula from Ilyaz et al. [27] to obtain at least 35 rats,

(1) E=total sample in groups × total groups−total groups,E=8 × 5−5,E=3520; if E > , the total sample is sufficient.

### 2.4. Generating the Obesity Rat Model

Selected rats were adapted for 7 days in group 4/cage under a 12‐h dark–light cycle, at 25°C–28°C, and 70% humidity at the Integrated Biology Laboratory, Universitas Sebelas Maret, Surakarta, Indonesia. The rats were given 20 g/day BR2 feed that each 100 g consisted of 310 kcal, 19%–20% crude proteins, 12% water, 5% fats, 5% crude fibers, 7% ashes, 0.8%–1.1% calcium, and 0.45% phosphorus, and drinking water ad libitum in adaptation periods. After adaptation, all male rats were given a 25 g/day high fat high fructose (HFHFr) and 10% fructose‐drinking water ad libitum for 28 days for obesity modeling. Compared to the standard diet during the adaptation period, the HFD in the treatment period provided approximately 1.9 times the caloric intake. Each 100 g of HFHFr consisted of 23 g BR2, 40 g beef fat, 12 g chicken liver, 28 g duck egg yolk, 4 g butter, and drinking high fructose water that consisted of 7 mL of 55% fructose syrup. The obesity modeling in this study followed the methods described in several previous studies and involved feeding rats 587.72 kcal of energy, 47.25 g of proteins, 52.61 g of fats, 32.25 g of carbohydrates, and 0.9 g of fiber [19, 28–30]. This HFHFr significantly increased BW, with mean ΔBW values of 51.00 ± 4.37 g and 79.80 ± 7.70 g after 30 days, in our previous findings [28, 30]. The rats’ BW and length were regularly measured using a digital weight scale and ruler. The Lee index (LI) was used to assess obesity in rats, with values of ≥ 300 g/cm^3^ considered indicative of obesity (Table 2).

**Table 2:** The average BW and Lee index of male rats before and after obesity induction.

Group		Mean ± SD		
		Before	After	Δ
BW (g)	NC	166.88 ± 10.26	252.00 ± 26.13	85.13 ± 21.20
	PC	165.89 ± 9.32	248.88 ± 35.27	83.00 ± 30.26
	BPL1	166.38 ± 7.91	248.13 ± 40.19	81.75 ± 33.36
	BPL2	165.25 ± 12.56	256.75 ± 33.47	91.50 ± 27.47
	BPL3	164.63 ± 10.65	251.00 ± 31.09	86.88 ± 29.37
	*p*	0.993^a^	0.987^b^	0.575^b^

Lee index (g/cm^3^)	NC	269.88 ± 8.78	307.39 ± 4.72	37.51 ± 5.76
	PC	276.33 ± 10.13	313.58 ± 7.98	37.25 ± 9.89
	BPL1	272.43 ± 10.78	308.03 ± 3.55	35.60 ± 10.80
	BPL2	273.01 ± 5.58	311.77 ± 6.43	38.76 ± 9.55
	BPL3	269.27 ± 3.70	305.95 ± 3.44	36.67 ± 5.80
	*p*	0.456^a^	0.067^a^	0.965^a^

*Note:* A significance threshold of *p* < 0.05 was used to determine statistical significance. The delta (Δ) values reflect the mean change before and after treatment for each group. Superscript letters (a, b) following the *p* values indicate the statistical tests applied to each parameter.

(a) One‐way ANOVA and (b) Kruskal–Wallis analyses with *p* < 0.05 as a significant difference.

### 2.5. Protocol of BPL Administration in Obese Male Rats

Once 40 male rats had LI > 300 g/cm^3^ after being induced with a HFD with 10% fructose, following the method of Kusumaningrum et al. [29], Devina et al. [28], Prasetyo et al. [19], and Sundari et al. [30] for 28 days, they were randomly allocated into five groups. In treatment periods, the negative control (NC) group received 20 g/day of BR2, while the PC group received 20 g/day of BR2 and 3 mL/day of PSE beverage. The treatment groups (BPL1, BPL2, and BPL3) were given 20 g/day of BR2 and 3 mL/day of BPL beverages at ratios of 75:25%, 80:20%, and 85:15%, respectively, for 21 days. The administration of BR2, which contained 310 kcal and ad libitum access to drinking water for 21 days during treatment, was intended to simulate a transition from HFHFr to a normal diet.

#### 2.5.1. Measurements of BW, LI, and BFC

Rats’ BW and length were measured weekly to evaluate the effects of BPL herbal beverage administration. The BW was recorded using a Joil digital scale with a maximum capacity of 5 kg and 1 g accuracy, while body length was measured from the naso to the anal using a ruler with 0.1‐cm precision.

The LI was calculated using the following formula:

(2) Lee index=BW13/ × 1000,Naso−anal length cm.

While BFC was determined using the following formula:

(3) BFC=0.58122.03 × Total mass index TMI−.

These methods were selected due to their practicality, nonreliance on sophisticated equipment, and proven correlation with direct measures of adiposity in our previous studies [28, 30].

#### 2.5.2. Measurement of Lipid Profile Levels

The lipid profiles, consisting of total cholesterol, LDL‐C, HDL‐C, and TG levels, were measured on Days 1, 14, and 21 of treatment. Rats were fasted for 6–8 h before taking blood samples in the morning on the following days. Blood samples were collected from the retro‐orbital plexus of each rat under light anesthesia using capillary tubes at baseline (Day 0) and after 21 days of treatment. Collected blood was centrifuged at 3000 rpm for 15 min to separate the serum. The reaction mixtures were incubated at room temperature (20°C–25°C) for 30 min before measurement. The cholesterol and LDL‐C levels were examined using the cholesterol oxidase peroxidase amino‐antipyrine (CHOD‐PAP) method, the HDL‐C levels were determined using the HDL‐C precipitate method, and the TG levels were determined using the glycerol peroxidase phosphate acid phenol amino phenazone (GPO‐PAP) method. All measurements of lipid profile were done using a Photometer Micro lab 300 at 546 nm wavelength at the Integrated Research and Testing Laboratory of Gadjah Mada University, Yogyakarta, Indonesia.

#### 2.5.3. Measurement of Leptin Levels and DPP4 Activity

Blood serum of all rats on Days 1 and 21 of treatment was used to measure the leptin levels, according to the protocol of the ELISA Kit Bioenzy, Indonesia Cat BZ‐08181650‐EB with sensitivity around 0.05 ng/mL. The assay demonstrated good precision with intra‐assay coefficients of variation (CV) of 3.20% and 4.06%, and an interassay CV of 4.52%. All values were well below the generally accepted thresholds (< 10% for intra‐assay and < 15% for interassay), indicating excellent repeatability and reproducibility of leptin measurements.

On the other hand, serum DPP4 activity was assessed using our previous study method [8]. In brief, 10 μL of rat serum samples was diluted with 40 μL of phosphate‐buffered saline (PBS), pH 7.4, and was mixed thoroughly with 50 μL of 2 mM Gly‐Pro *p*‐nitroanilide substrate (Sigma‐Aldrich, St. Louis, MO, USA). The sample absorbance was measured using a spectrophotometer at 405 nm for 60 min at 25 °C. DPP4 activity was then calculated using the Beer–Lambert formula, A = εCl, where A is the absorbance, *ε* is the μmolar extinction coefficient (9.45 L·μmol^−1^ cm^−1^ for pNA at 405 nm), C is the concentration (μmol·litre^−1^), and *l* is the length of the light path. The intra‐assay CV for DPP4 activity was 3.52%, while the interassay CV was 9.20%.

### 2.6. Statistical Analysis

All collected data were analyzed using the Statistical Program for Social Science (SPSS) 27.1 (the International Business Machines Corporation, USA). Data normality was assessed using the Shapiro–Wilk test with *p* > 0.05. All numeric data were presented as mean ± standard deviation (SD). The one‐way analysis of variance (ANOVA) and least significant difference (LSD) post hoc tests were used to compare the mean of LI, BFC, LDL‐C, HDL‐C, and leptin levels, and DPP4 activity among groups. Meanwhile, the Kruskal–Wallis and Mann–Whitney tests were applied to compare BW, total cholesterol, and TG levels among groups. In addition, repeated‐measures ANOVA was performed to assess changes in the BMI, LI, and BFC before, during, and after treatment. Significant values of all statistical analyses were set at *p* < 0.05.

## 3. Results

### 3.1. The BPL Herbal Beverages Reduced BW in Male Rats With Obesity

At first, we evaluated the effects of BPL herbal beverages on the BW as a nutritional status of male rats with obesity. Figures 1(a), 1(b) show that BPL beverages reduced the average BW in a dose‐dependent manner. At baseline, the average BW did not differ significantly among all groups (*p* = 0.987). During the intervention period (Days 7 and 14), the BPL1–BPL3 groups showed a significant decrease in BW compared with the NC group (*p* = 0.013; *p* < 0.001; and *p* < 0.001, respectively), although the reductions at Day 7 were not statistically significant. By Day 21, the BW reductions became significant in the BPL2 (38.88 ± 15.58 g; *p* = 0.001) and BPL3 groups (42.38 ± 10.06 g; *p* < 0.001) compared with the NC group. In addition, the BW in the BPL3 group was significantly lower than in the PC group (*p* = 0.034). No significant differences were observed among the treated BPL groups during 21‐day treatment (*p* = 0.803, *p* = 0.596, and *p* = 0.638, respectively).

Figure 1The average BW in five rat groups with or without BPL herbal beverage administrations (a‐b). The NC and PC groups were given 20 g/day BR2 only and BR2+3 mL/day PSE beverage, respectively. The BPL1, BPL2, and BPL3 groups were given 20 g/day BR2 and 3 mL/day 75:25%, 80:20%, and 85:15% BPL beverages for 21 days. All treatments were administered orally once daily at 9 a.m. (^∗^) represents a significant difference among the rat groups with *p* < 0.05. (^∗∗^) represents a significant difference before, during, and after treatment with *p* < 0.05.

**Figure:**
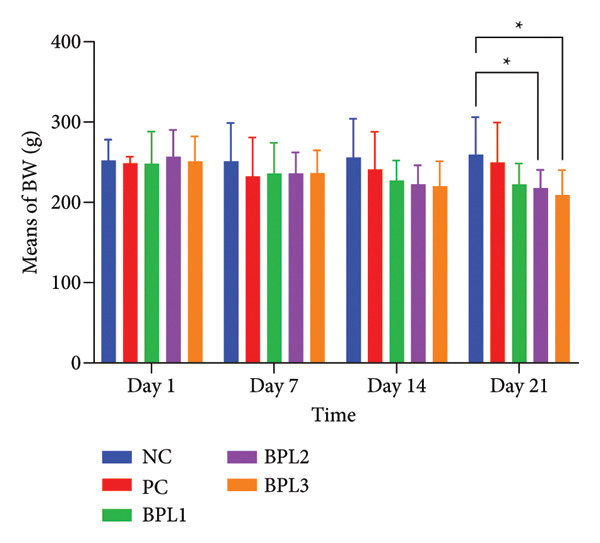
(a)

**Figure:**
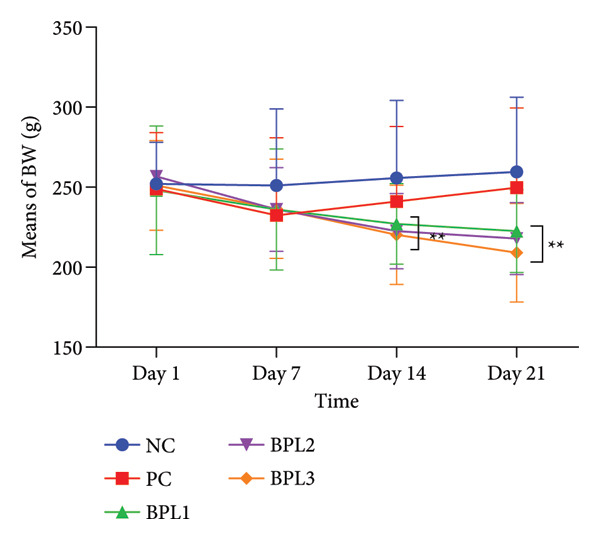
(b)

### 3.2. The BPL Herbal Beverages Reduced the LI and the BFC Percentage in Male Rats With Obesity

The next evaluation of nutritional status changes in male rats treated with BPL herbal beverages was LI and BFC (Figures 2(a), 2(b), 2(c), 2(d)). At baseline, the average LI in the PC and BPL2 groups was higher than in the other groups but not significantly different (*p* = 0.067). Figures 2(a), 2(b) show LI in the NC group slightly increased (from 307.39 ± 4.72 to 308.85 ± 13.45 g/cm^3^), and the PC group slightly decreased (from 313.58 ± 7.98 to 312.33 ± 15.14 g/cm^3^) during the BPL herbal beverage treatment. On Days 7 and 14, all groups except the NC group exhibited lower LI compared to baseline, with the greatest reductions observed in BPL1–BPL3 groups (11.41 ± 5.85, 16.35 ± 5.13, and 18.43 ± 4.89 g/cm^3^, respectively). The average LI in BPL1–BPL3 groups on Day 21 was significantly lower than the NC (*p* = 0.024; 0.014; and 0.001, respectively).

Figure 2The Lee index (a) and (b) and BFC (c) and (d) in male rats with obesity treated with the BPL herbal beverages. The NC and PC groups were given 20 g/day BR2 only and BR2+3 mL/day PSE beverage, respectively. The BPL1, BPL2, and BPL3 groups were given 20 g/day BR2 and 3 mL/day 75:25%, 80:20%, and 85:15% E beverages for 21 days. (^∗^) represents a significant difference among the rat groups with *p* < 0.05. (^∗∗^) represents a significant difference before, during, and after treatment with *p* < 0.05.

**Figure:**
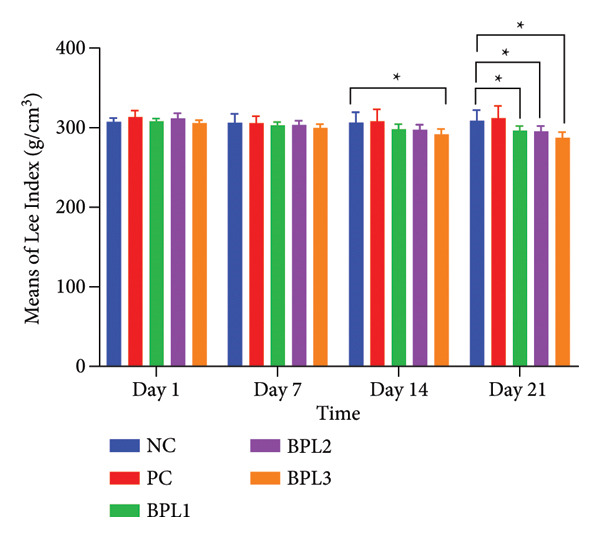
(a)

**Figure:**
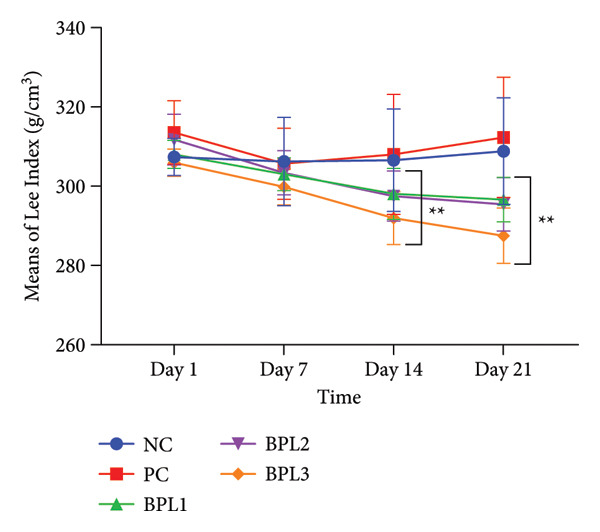
(b)

**Figure:**
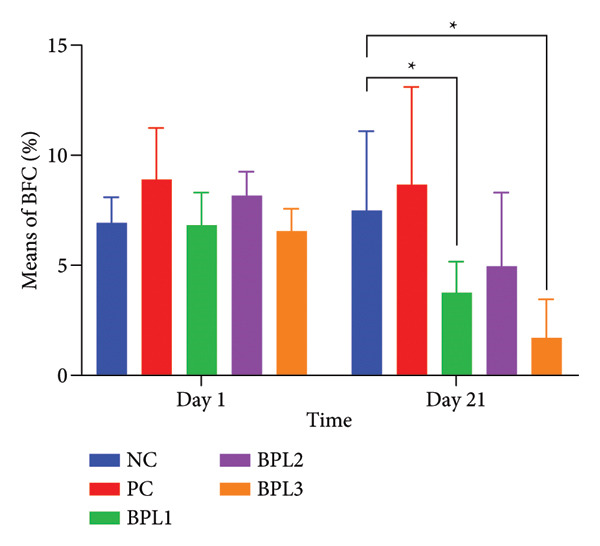
(c)

**Figure:**
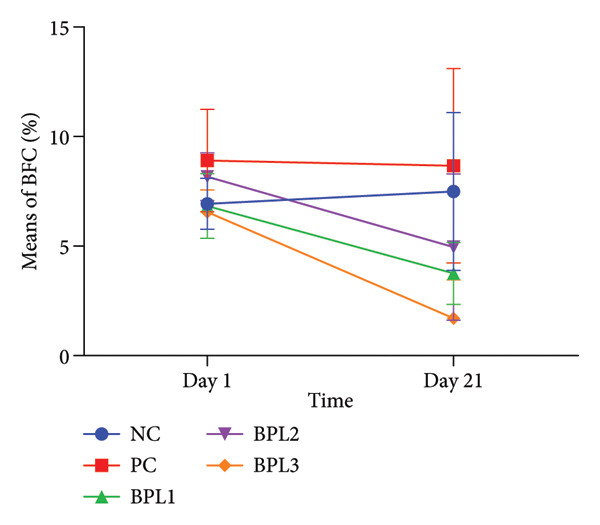
(d)

The BFC percentage was calculated among rat groups before and after BPL herbal beverage treatment (Figures 2(c), 2(d)). At baseline, the PC and BPL2 groups had higher BFC percentages than the NC group, although only the PC group differed significantly. On Day 21, the BPL3 showed the greatest reduction in the BFC percentage (4.83 ± 1.13%) compared to the NC (*p* = 0.001) and PC groups (*p* < 0.001). The average BFC percentages in the BPL1 and BPL3 groups were significantly lower than those of the NC group (*p* = 0.001 and < 0.001, respectively).

### 3.3. The BPL Herbal Beverages Had Different Effects on Lipid Profiles in Male Rats With Obesity

We further evaluated lipid profiles in male obese rats treated with the BPL herbal beverage (Figures 3(a), 3(b), 3(c), 3(d)). Figure 3(a) shows that BPL1 reduced total cholesterol levels but the decrease was not significantly different from the NC and PC groups. From Figure 3(b), the average LDL‐C levels decreased in the BPL1 and BPL3 groups, but it was not significantly different compared to the NC and PC groups (*p* = 0.354 and *p* = 0.370). At baseline, the average LDL‐C levels in the NC and BPL1 groups were higher than those of other rat groups, but it was not significantly different (*p* = 0.704). During treatment, average LDL‐C levels increased in all rat groups on Day 14, but LDL‐C reductions were observed on Day 21 in the NC, PC, and BPL1 groups. On Day 21, reductions in LDL‐C were shown in the BPL1 group, but it did not differ from the other groups (*p* = 0.438, 0.340, and 0.845).

Figure 3The averages of total cholesterol levels (a), LDL‐C levels (b), HDL‐C levels (c), and triglyceride levels (d) in male rats with obesity treated with the BPL herbal beverages. The NC and PC groups were given 20 g/day BR2 only and BR2+3 mL/day PSE beverage, respectively. The BPL1, BPL2, and BPL3 groups were given 20 g/day BR2 and 3 mL/day 75:25%, 80:20%, and 85:15% BPL beverages for 21 days (^∗^) represents a significant difference among the rat groups with *p* < 0.05.

**Figure:**
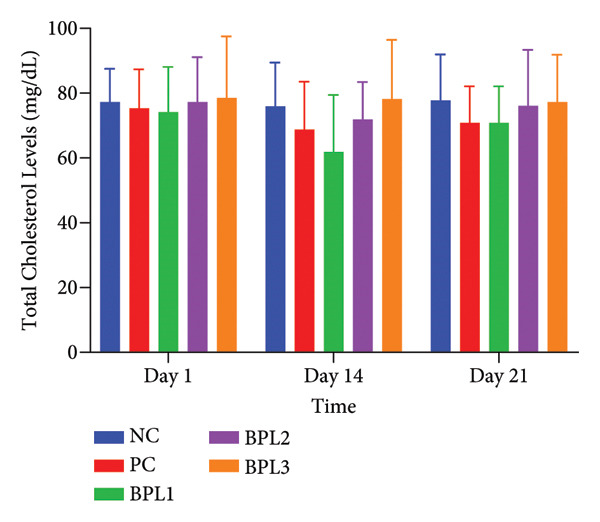
(a)

**Figure:**
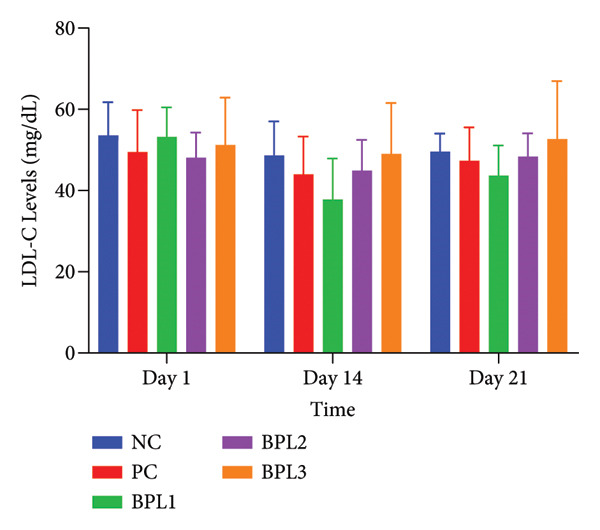
(b)

**Figure:**
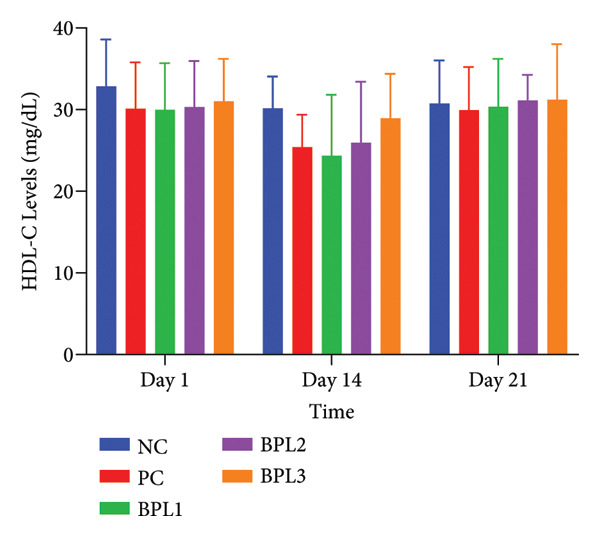
(c)

**Figure:**
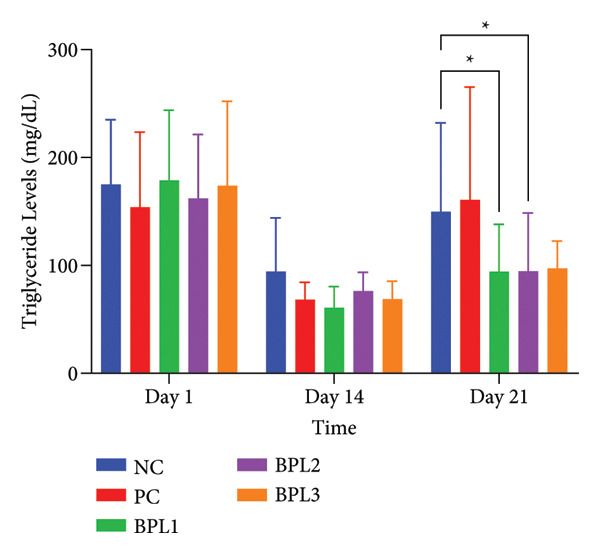
(d)

Figure 3(c) shows that at baseline, the average HDL‐C levels in the NC and BPL2 groups were higher than in the other groups but not significantly different (*p* = 0.836). At Day 14 treatment, HDL‐C levels decreased in all groups, followed by a rebound increase on Day 21. After the treatment period, the average HDL‐C levels in the NC and BPL1–BPL3 groups increased compared with baseline.

TG levels have a similar trend to HDL‐C after BPL treatment (Figure 3(d)). The average TG levels in the NC and BPL1 groups were higher than in the other groups but not significantly different (*p* = 0.949) at the beginning of treatment. Average TG levels decreased in all rat groups on Day 14 but then increased again on Day 21. Significant reductions in TG levels were observed in the BPL1 and BPL2 groups compared with the NC group (*p* = 0.021 and *p* = 0.027, respectively). At the end of BPL treatment, TG levels in the BPL1–BPL3 groups were lower than at baseline but did not differ significantly among the treatment groups themselves (*p* = 0.529, 0.529, and 0.248).

### 3.4. The BPL Herbal Beverages Reduced the Leptin Levels of Male Rats With Obesity

**Figure 4:**
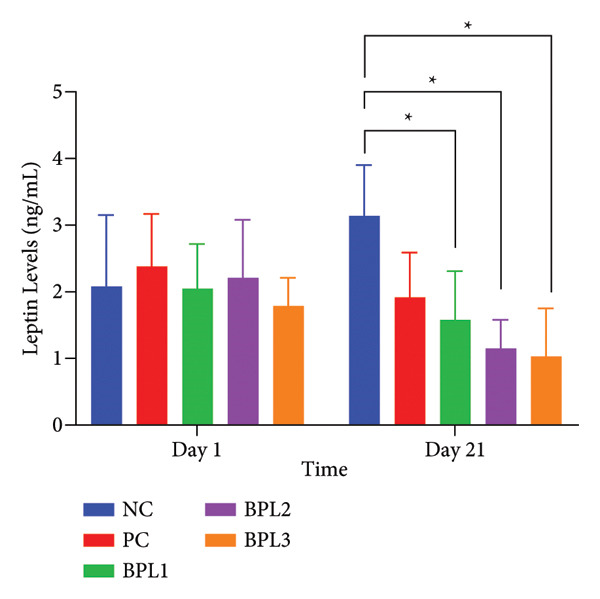
The leptin level changes in five groups with or without BPL herbal beverage administration. The NC and PC groups were given 20 g/day BR2 only and BR2+3 mL/day PSE beverage, respectively. The BPL1–BPL3 groups were given 20 g/day BR2 and 3 mL/day 75:25%, 80:20%, and 85:15% BPL beverages for 21 days (^∗^) represents a significant difference among the rat groups with *p* < 0.05.

Leptin levels were assessed to determine the effects of BFL herbal beverages on male rats with obesity (Figure 4). At Day 1 of treatment, the BPL3 group had the lowest leptin levels, but the average levels among all groups did not differ significantly (*p* = 0.658). During treatment, leptin levels decreased in the PC and BPL1–BPL3 groups, whereas the NC group exhibited a significant increase in average leptin levels before and after treatment (*p* = 0.007). On Day 21, the BPL2 and BPL3 groups showed lower leptin levels (1.06 ± 0.86 and 1.03 ± 0.72 ng/mL, respectively) than the PC and BPL1 groups. Compared with the NC and PC on Day 21, the average leptin levels in the BPL2 and BPL3 groups were significantly lower. However, no significant differences in leptin levels were found among the BPL1–BPL3 groups (*p* = 0.218, 0.112, and 0.708, respectively).

### 3.5. The BPL Herbal Beverages Increased the DPP4 Activity of Male Rats With Obesity

**Figure 5:**
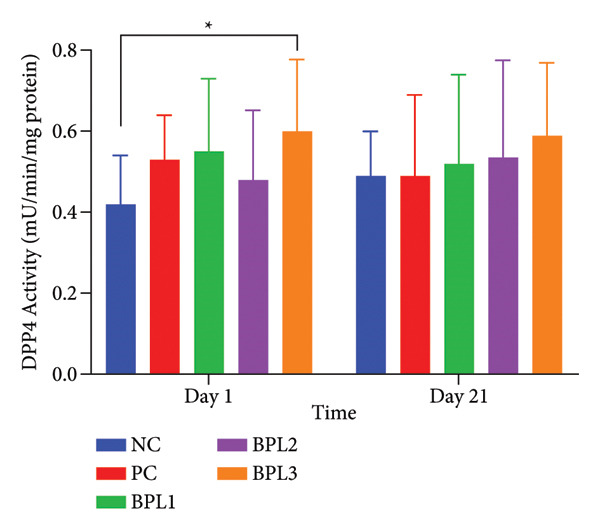
The DPP4 activity changes in five groups with or without beverage administration. The NC and PC groups were given 20 g/day BR2 only and BR2+3 mL/day PSE beverage, respectively. The BPL1–BPL3 groups were given 20 g/day BR2 and 3 mL/day 75:25%, 80:20%, and 85:15% BPL beverages for 21 days (^∗^) represents a significant difference among the rat groups with *p* < 0.05.

Another adipokine, DPP4 activity, was also evaluated in male obese rats with the BPL treatment (Figure 5). The NC group showed the lowest DPP4 activity, which was significantly different from the BPL3 group (*p* = 0.028) but not from the other groups (*p* = 0.212) on Day 1. During treatment, increased DPP4 activity was observed across all treatment groups. On Day 21, the BPL3 group had the highest DPP4 activity (0.589 mU/min/mg protein) compared with the NC and PC groups, but not significantly different (*p* = 0.707 and *p* = 0.865, respectively).

## 4. Discussion

In the present study, the administration of BPL herbal beverages in all combinations containing phytochemicals and low energy demonstrated to reduce BW, LI, BFC, TG, and leptin levels in male rats with obesity. The BPL3 beverage with an 85:15% ratio of butterfly pea flowers and lemon juice is the best herbal drink in reducing BW (42.38 ± 10.06 g), LI (18.43 ± 4.89 g/cm^3^), and BFC (4.83 ± 1.13%). The BPL3 group achieved the greatest BW reduction by 16.87%, followed by BPL2 with 15.14% and BPL1 with 10.32%. However, TG levels were greatly reduced during the BPL beverage treatment, with the highest reduction in BPL1 by 47.1%, followed by BPL3 with 44% and BPL2 with 41.7%. The greatest reduction in leptin levels occurred in the BPL2 group by 47.9%, followed by the BPL3 group with 42.4% and the BPL1 group with 22.9%, which is similar to the PSE administration (19.3%). Surprisingly, the BPL beverages did not affect cholesterol profiles and DPP4 activity. Altogether, our findings suggest that BPL3 and BPL2 might reduce BW and BFC through the inhibition of lipid metabolism and leptin secretion.

According to the WHO and World Obesity Federation guidelines, an effective intervention for obesity management should achieve at least a 10% BW reduction from baseline [1, 31]. This in vivo study clearly indicates that the BPL beverages match that criterion and become a potential herbal beverage for obese individuals. Our findings are in accordance with previous studies that used butterfly pea flower extract or lemon juice only. The administration of 1000 mg of butterfly pea flower extract for 21 days in anxiety model mice reduced BW by only ±16.39 g [13]. In contrast, Hamsi [32] found that administration of 1 mL/day of lemon juice for 21 days led to a BW reduction of approximately 42.63 g in male Wistar rats with HFD, similar to our BPL3 beverage. The BW reduction in our study may be attributed to the synergistic antioxidant and metabolic‐modulating properties of butterfly pea flowers and lemon juice, which could enhance the efficacy of dietary interventions in reducing adiposity and improving metabolic health. The BPL1–BPL3 formulations have high levels of total flavonoids (993.50 ± 6.36 to 1265.00 ± 21.92 ppm) and anthocyanins (424.41 ± 25.75 to 485.38 ± 22.08 ppm), demonstrating that all beverage combinations provide substantial amounts of bioactive compounds. In a previous study, butterfly pea flower demonstrated the reductions in multiple metabolic syndrome parameters, such as improving lipid profile, blood glucose, SOD, and CAT in rats with a high‐fat, high‐carbohydrate diet [33]. In addition, lemon extract improved insulin signaling and reduced inflammatory cytokines in cultured adipocytes [34]. In our study, adding lemon juice significantly increased the antioxidant activity of the BPL compared to butterfly pea flower alone. Therefore, it indicates that the mixture of butterfly pea flowers and lemon fruits has strong antioxidant activity [35, 36].

The administration of BPL herbal beverages also reduces the LI in male obese rats, whereas the PSE‐containing drink increases the LI. Although the rats in the PC group experienced a decrease in BW and LI on the seventh day of intervention, the remaining days revealed a progressive increase (Figures 1 and 2). The existing evidence suggests that PSE’s administration has inconsistent BW loss [37]. A randomized controlled trial in postmenopausal women indicated that daily consumption of 3000 mg PSE for seven weeks significantly reduced serum cholesterol in normal weight, overweight, and obese participants. This finding emphasizes that PSE could reduce cholesterol levels among participants with different BMIs but not BW reduction. Moreover, its ability to reduce BW or adiposity indices, such as the LI, appears minimal, particularly in obese models [38].

The higher the flavonoid and anthocyanin contents in the BPL beverages, the greater the effect of BW and LI reductions in obese rats is. Delphinidin is an anthocyanidin derivative that is abundant in butterfly pea flowers. It can activate AMPK and increase adipose triglyceride lipase (ATGL) activity, which accounts for approximately 90% of lipolytic activity in adipose tissues [39, 40]. In addition, delphinidin downregulates the expression of peroxisome proliferator–activated receptor gamma (PPAR‐γ), thereby suppressing adipogenesis and further contributing to body fat reduction [41]. Recent in vivo evidence indicates that anthocyanins such as Cyanidin‐3‐O‐glucoside and Delphinidin‐3‐O‐glucoside could restore mitochondrial biogenesis via PPAR‐γ, PR domain containing 16 (PRDM16), and PPAR‐γ Coactivator 1‐alpha (PGC‐1*α*). It also enhances thermogenesis via Uncoupling Protein 1 (UCP‐1) and maintains mitochondrial dynamics in obese rats through the modulation of the Protein Kinase A, AMPK, and Sirtuin 1 signaling pathways [42]. These mechanistic actions likely explain the superior efficacy of BPL beverages in reducing both BW and LI in male rats with obesity. Alkaloid contents in lemon fruits are unknown [20], but orange fruits contain alkaloid synephrine, which prevents weight gain and adiposity by modulating amino acid metabolism and reducing adipose inflammation in mice with HFD [43]. In addition, hesperidin is a major citrus flavonoid that can activate AMPK and PPAR‐α signaling. These actions result in reducing hepatic lipid accumulation, improving insulin sensitivity, suppressing lipogenesis, and stimulating CCK to reduce appetite [44, 45].

To further investigate how the BPL beverages could reduce BW and LI in male obese rats, we assessed TG and leptin levels and DPP4 activity. The higher amount of lemon juice in the BPL herbal beverage has a higher effect on the reduction in TG levels. Although we did not perform the administration of the butterfly pea flower extract only on TG levels of male obese rats, the administration of roselle flower extract, which shares similar anthocyanin content, could reduce TG levels in rats with dyslipidemia [46]. Besides alkaloids and flavonoids, lemon juice also comprises saponin, which could reduce TG levels by inhibiting gut fat absorption through intestinal permeability [45, 47]. The reduction in TG levels may also relate to switching from an HFHFr to a standard feed, too.

In addition to the reduction in TG levels, BPL herbal beverages can reduce leptin levels. While no prior studies have directly examined butterfly pea flower extract and the BPL herbal beverages on leptin levels, they have been shown to reduce adipogenesis, which may lower leptin production since adipocytes are the primary leptin source [7, 48, 49]. Several studies have reported that anthocyanins in butterfly pea flower extract enhance insulin sensitivity by regulating adiponectin, which influences insulin and leptin secretion, thereby improving leptin sensitivity and appetite control [7, 50]. Similarly, hesperidin in lemon juice reduced serum leptin in rats given HFD [48]. Besides flavonoids and anthocyanins, phenols and saponins in the BPL herbal beverage are likely to contribute to serum leptin reduction. Conversely, rats fed only a standard diet showed increased leptin, suggesting that calorie restriction without fat reduction does not improve leptin sensitivity. The lack of weight and fat mass reduction in the NC group align with leptin secretion being proportional to adipose tissue. Leptin resistance can raise circulating leptin, disrupting hypothalamic regulation and promoting excessive energy intake [51].

Unfortunately, our study did not show that BPL herbal beverages affect serum DPP4 activity. Our results are different from a previous study that reported administration of 80 mg/kg BW/day teneligliptin (DPP4 inhibitor) prevented obesity in mice given HFD for 10 weeks [52]. Another study indicated that oral administration of 250 mg/kg BW of *Anogeissus latifolia* extract could inhibit DPP4 activity in male rats with obesity and glucose intolerance [53]. The inability of BPL herbal beverages to inhibit DPP4 activity may indicate that their anti‐obesity effects probably pass through other mechanisms, which inhibit TG absorption and leptin secretion. These findings highlight further research on optimizing formulations of BPL herbal beverages or combining with more potent DPP4 inhibitors.

This study has several limitations that should be acknowledged. The duration of the BPL herbal beverage intervention was short, only 21 days, which may not fully capture the long‐term effects or sustainability of weight loss and metabolic improvements. More detailed parameters, such as fat mass distribution, adipocyte histology, adiponectin levels, and gene expression related to lipid metabolism (e.g., PPAR‐γ, AMPK, and ATGL), and detailed bioactive compounds, such as hesperidin and delphinidin, were not assessed, which limits the understanding of the mechanistic basis of the BPL herbal beverage. Lastly, the study did not include a group receiving only butterfly pea extract or only lemon beverage, making it difficult to delineate the individual contributions of each component in the combined formulation.

## 5. Conclusion

The BPL herbal beverage demonstrated formulation‐specific effects on nutritional status, lipid profiles, and adipokines in male rats with obesity. Administration of the BPL herbal beverage at an 85:15% ratio significantly reduced BW and LI in male rats with obesity. The formulation of the BPL with the 80: 20% and 85:15% ratios has the greatest decrease in leptin levels. None of the BPL herbal formulations altered cholesterol metabolism or DPP4 activity, indicating a selective activity on lipid and endocrine parameters. Overall, the BPL herbal beverage with an 85:15% ratio exerts its anti‐obesity effect through the inhibition of TG absorption and leptin secretion.

In future studies, the quantification and characterization of bioactive compounds in butterfly pea flower extract, lemon juice, and their combination are required for phytochemical profiling and targeted mechanistic testing to confirm their specific contributions. Further research is strongly recommended to explore the underlying mechanisms of action and molecular interactions of the BPL herbal beverages, particularly in relation to other adipokines, inflammation, and glucose metabolism pathways. Long‐term studies are necessary to assess the safety, bioavailability, and sustained efficacy of each formulation. Special attention for 85:15% formulation, it promises to improve both lipid parameters and endocrine function related to obesity.

## Conflicts of Interest

The authors declare no conflicts of interest.

## Funding

This study was supported by Universitas Sebelas Maret, Research Grants 194.2/UN27.22/PT.01.03/2024 and 369/UN27.22/PT.01.03/2025.

## Data Availability

The data that support the findings of this study are available on request from the corresponding author. The data are not publicly available due to privacy or ethical restrictions.
